# Application of the Extended Theory of Planned Behavior to Predict Exclusive Breastfeeding Intention, In Pregnant Nulliparous Women. A Cross-Sectional Study

**DOI:** 10.17533/udea.iee.v40n2e04

**Published:** 2022-09-15

**Authors:** Farahnaz Yazdanpanah, Mostafa Nasirzadeh, Hassan Ahmadinia, Mahdi Abdolkarimi

**Affiliations:** 1 M.Sc. student in Health Education and Health Promotion, Department of Health Education and Health Promotion, School of Health, Student Research Committee, Rafsanjan University of Medical Sciences, Rafsanjan, Iran. Email: farahnazyazdanpanah.f11@gmail.com Iran University of Medical Sciences Department of Health Education and Health Promotion School of Health University of Medical Sciences Rafsanjan Iran farahnazyazdanpanah.f11@gmail.com; 2 Assistant Professor, Department of Health Education and Health Promotion, School of Health, Occupational Health and Safety Research Center, NICICO, World Safety Organization and Rafsanjan University of Medical Sciences, Rafsanjan, Iran.Email: mnasirzadeh13@yahoo.com Iran University of Medical Sciences Department of Health Education and Health Promotion School of Health, Occupational Health and Safety Research Center Rafsanjan University of Medical Sciences Rafsanjan Iran mnasirzadeh13@yahoo.com; 3 PhD of Biostatic, Dept. Of Epidemiology and Biostatics, Medical School, Rafsanjani University of Medical Sciences, Rafsanjan, Iran.Email: h.ahmadinia@gmail.com Iran University of Medical Sciences Dept. Of Epidemiology and Biostatics Medical School Rafsanjani University of Medical Sciences Rafsanjan Iran h.ahmadinia@gmail.com; 4 Assistant Prof. Dept. of Health Education and Health Promotion, School of Health, Rafsanjan University of Medical Sciences, Rafsanjan, Iran. Email: mahdi_13581@yahoo.com. Iran University of Medical Sciences Dept. of Health Education and Health Promotion School of Health Rafsanjan University of Medical Sciences Rafsanjan Iran mahdi_13581@yahoo.com

**Keywords:** pregnant women, breast feeding, self efficacy, social support, health behavior, mujeres embarazadas, lactancia maternal, autoeficacia, conductas relacionadas con la salud, gestantes, aleitamento materno, autoeficacia, apoio social, comportamentos relacionados com a saúde.

## Abstract

**Objective.:**

This study investigated the effect of Extended Theory of Planned Behavior (ETPB) extended theory of planned behavior in comparison with the Theory Of Planned Behavior (TPB) in explaining the intention of Exclusive Breastfeeding Intention (EBF) in Pregnant nulliparous women of Kerman (Iran).

**Methods.:**

In this descriptive study, 249 pregnant women in Kerman participated via simple random sampling. The research instruments included Questionnaire related to the structures of the theory of planned behavior, breastfeeding self-efficacy and social support questionnaire for breastfeeding.

**Results.:**

The results of the correlation test showed a significant relationship between all constructs of the theory of extended planned behavior and the intention of EBF. The highest correlation belonged to the construct of subjective norms (r=0.49). Path regression coefficients in the second model showed that the Self-Efficacy mediator variable is fully capable of meaningful mediation between Social Support and Intention (*p*<0.001; B=0.383). The conceptual diagram of Structural equation modeling showed a higher explained variance or R2 index for the intention variable for the developed model compared to that of the first model, i.e. (the first model: R2=0.37, the second model: R2=0.46). The goodness-of-fit index had a better status for the developed model.

**Conclusion.:**

Extended TPB with social support and breastfeeding self-efficacy constructs can be appropriate model for predicting the intention and behavior of EBF.

## Resumen

**Objetivo.:**

Este estudio investigó el efecto de la Teoría Extendida del Comportamiento Planificado (TECP) en comparación con la Teoría del Comportamiento Planificado (TCP) para explicar la intención de la Lactancia Materna Exclusiva (LME) en mujeres nulíparas embarazadas de Kerman.

**Métodos.:**

En este estudio descriptivo participaron 249 mujeres embarazadas de la ciudad de Kerman (Iran), seleccionadas por muestreo aleatorio simple. Los instrumentos de investigación incluían un cuestionario relacionado con las estructuras de la teoría de la conducta planificada, un cuestionario de autoeficacia para la lactancia materna y un cuestionario de apoyo social para la lactancia materna.

**Resultados.:**

La prueba de correlación mostró una relación significativa entre todos los constructos de la TECP y la intención de LME. La correlación más alta correspondió al constructo de normas subjetivas (r=0.49). Los coeficientes de la regresión en el segundo modelo mostraron que la variable Autoeficacia mediaba significativamente entre las variables Apoyo Social e Intención (*p*<0.001; B=0.383). El diagrama conceptual del Structural equation modeling mostró una mayor varianza explicada o índice R2 para la variable de intención para el modelo desarrollado en comparación con el del primer modelo, es decir, (el primer modelo: R2=037, el segundo modelo: R2=0.46). El índice de bondad de ajuste fue mejor para el modelo desarrollado.

**Conclusión.:**

La TECP con los constructos de apoyo social y autoeficacia para la lactancia materna puede ser un modelo apropiado para predecir la intención y el comportamiento de la LME.

## Resumo

**Objetivo.:**

Este estudo investigou o efeito da Teoria Estendida do Comportamento Planejado (TECP) comparada à Teoria do Comportamento Planejado (TCP) na explicação da intenção da Lactância Materna Exclusiva (LME) em gestantes nulíparas de Kerman.

**Métodos.:**

Neste estudo descritivo, participaram 249 gestantes da cidade de Kerman (Irã), selecionadas por amostragem aleatória simples. Os instrumentos de pesquisa incluíram um questionário relacionado às estruturas da teoria do comportamento planejado, um questionário de autoeficácia para amamentar e um questionário de apoio social à amamentação.

**Resultados.:**

O teste de correlação mostrou uma relação significativa entre todos os construtos do TCP e a intenção de LME. A maior correlação correspondeu ao construto das normas subjetivas (r=0.49). Os coeficientes de regressão no segundo modelo mostraram que a variável Autoeficácia mediou significativamente entre as variáveis Apoio Social e Intenção (p <0.001; B=0.383). O diagrama conceitual da Estrutura equação a modelagem apresentou maior variância explicada ou índice R2 para a variável intenção para o modelo desenvolvido em relação ao primeiro modelo, ou seja, (o primeiro modelo: R2=0.37, o segundo modelo: R2=0.46). O índice de bondade de ajuste foi melhor para o modelo desenvolvido.

**Conclusão.:**

A TECP com os construtos de apoio social e autoeficácia em amamentar pode ser um modelo adequado para predizer a intenção e o comportamento do LME.

## Introduction

The World Health Organization (WHO) defines EBF as “giving no other food or drink -not even water- except breast milk” and the medications, such as oral therapies, vitamins, and supplements.^1^ Breast milk plays a vital role in preventing gastrointestinal and respiratory infections.^2^ It also contributes to maternal health and well-being.^3^ However, a low rate of EBF has been reported even in developed countries.^4^ At its 65^th^ meeting, the (WHO) decided to increase the rate of EBF to at least 50% by 2025. In developing countries, the concerning rate is 39%, and in Iran, it varies between 13% and 77%.^5^ A study conducted in Riyadh found that although participants had a breastfeeding rate of 72%,^4^ only %20.9 had had EBF for six months.^6^

Early cessation of breastfeeding causes irreparable physical, mental, and socio-economic damages to the child, and ultimately, society.^7^ Subjective norms in lactating and postpartum women, including the views of significant individuals, such as coevals and social networks or important family members, such as partner, parents, or siblings, are important factors in breastfeeding.^8^ One of the effective theories in changing behaviors influenced by social norms is (TPB), which was developed in 1991 by Ajzen and Fishbein and has the following constructs: Behavioral intent, Attitude toward behavior, Subjective norms And Perceived behavioral control.^9^

TPB is a behavioral theory in the field of social psychology, which analyzes the factors affecting behavioral goals and explains the behavior. The theory notes that the main predictor of behavior is a change in the behavioral intention that depends on the attitudes, subjective norms, and the perceived behavioral control of individuals.^10^ This theory, which has been used for many health behaviors, explains on average about 40% of the relationships between the intent and health behaviors.^11^ Some researchers believe that for some behaviors, such as successful breastfeeding, using the concept of self-efficacy is better than the perceived behavioral control variable because it makes it possible to predict the occurrence of the behavior. Breastfeeding self-efficacy is a social cognitive theory adapted from Dennis. Breastfeeding self-efficacy reflects how a mother perceives her breastfeeding ability rather than her true ability to succeed in breastfeeding. Mothers with high self-efficacy can often overcome the barriers that seem exhausting to the mothers with low self-efficacy.^12^ And this is a modifiable factor that can affect breastfeeding success.^13^ One critique of the TPB is neglecting the social factors affecting behavior. Numerous studies have emphasized the role of social support and especially the spouses’ attention to the initiation and continuation of EBF. Breastfeeding is a behavior that requires mothers’ knowledge, skills, support, and confidence.^14^ Social factors, including the support of social groups, affect the mothers’ breastfeeding.^15^ Therefore, considering the importance and role of EBF in promoting the health of infants and children, the present study aimed to investigate the predictive power of the TPB constructs in explaining the intention of EBF in pregnant nulliparous women of Kerman and compare it with an extended theory, that in addition to the constructs of the TPB, also includes the self-efficacy constructs of breastfeeding and social support.

## Methods

This is a descriptive cross-sectional study in which 249 pregnant nulliparous women referring to comprehensive health service centers in Kerman in November and December 2020 are included in the study. The criteria for inclusion in the study were participants' willingness and being nulliparous, and the exclusion criteria of the study were being infected with breast diseases that prohibit breastfeeding and diseases the treatment of which interferes with breastfeeding. Some statisticians recommend a minimum sample size of 200 in SEM studies.^16^ Considering the probable loss data, the final sample size was 249. The samples were also homogenized in terms of participation in childbirth preparation classes and in terms of behavioral intent. Written informed consent was obtained from the participants. 

Sampling was conducted through the simple random sampling method. After coordination with the officials of Kerman health center, six public centers of comprehensive health services were randomly selected, and in each center, the samples were included in the study based on the electronic file of the pregnant women and based on inclusion and exclusion criteria. The instrument used in the research was a questionnaire consisting of four sections that included a section to record the demographic characteristics, such as age, education, and occupation, a TPB model structures assessment questionnaire, taken from Alami *et al* study. 17 In the form of 25 five-choice questions, on a Likert scale (attitude in the form of 11 questions, subjective norms in the form of seven questions, perceived behavioral control in the form of four questions, and behavioral intention in the form of three questions), Benson’s breastfeeding behavior self-efficacy questionnaire,^18^ and breastfeeding social support questionnaire taken from the Boateng study^19^, In the form of 14 tree-choice question.

In the constructs section of the TPB, the questions were assessed using a five-point Likert scale. In case of complete agreement (strongly agree), a score of five, and in the case of disagreement (strongly disagree), a score of one was given to the relevant question. The instrument related to model constructs has acceptable content validity (0.66 to 0.99), and Cronbach's alpha coefficient and intra-class correlation coefficient are 0.79 and 0.81, respectively.^17^. The self-efficacy breastfeeding questionnaire consisted of 13 questions. The findings of content and face validity showed almost perfect results. In the reliability study, Cronbach's alpha was also evaluated as favorable (0.91).^18^ Also, in the present study, Cronbach's alpha coefficient was recalculated for self-efficacy questions, which was equal to 0.85.

In this study, the breastfeeding social support scale was used, the validity and reliability of which were confirmed.^19^ Its validity was re-evaluated qualitatively using the experts 'opinions and determining the CVR and CVI indices. In the reliability study, Cronbach's alpha coefficient was calculated as 0.84. Based on the validity indicators, the questionnaire was approved with 13 questions. Finally, the data were entered into SPSS (version 22) (IBM, Armonk, NY, USA).Pearson correlation coefficient was used to determine the correlation between them according to the normal distribution. Independent t-tests and one-way analysis of variance (ANOVA) were used to compare the scores according to demographic characteristics and independent variables of the study. The concept diagram of SEM was used to compare the two models. This research was supported by Rafsanjan University of Medical Sciences. Conscious consent was obtained from all samples to participate in the study. Ethical approval for this study was approved by Research Ethics Committee with the ethics code of IR.RUMS.REC.1399.102.

## Results

A total of 249 mothers were studied in this research. The age range of pregnant mothers was 15-43 years, with an average of 26.98 years. The majority of the women participating in this study (90%) were housewives. Also, the majority of them had a high school diploma or higher (44.2% university education and 35.7% high school diploma. Among the demographic characteristics, the independent t-test statistic showed a significant difference between the mean scores of attitude (*p*=0.033, t=-2.138), self-efficacy (p=0.005, t=-2.803) and behavioral intention (*p*=0.021, t=-2.323) by age. The results of the Pearson correlation test showed a statistically significant relationship between all constructs of the extended TPB and the intention of EBF (Table 1).

**Table 1:** Correlation matrix between the constructs of the ETPB and intention for EBF

	Mean ± SD	Attitude	Subjective Norm	Perceived Control	Self-Efficacy	Social Support	Intention
Attitude	44.77±4.40	1					
Subjective Norm	30.97±3.53	0.49*	1				
Perceived Control	11.46±2.20	0.45*	0.44*	1			
Self-Efficacy	13.11±1.90	0.28*	0.42*	0.19*	1		
Social Support	51.17±8.35	0.25*	0.25*	0.27*	0.25*	1	
Intention	35.93±6.53	0.34*	0.54*	0.28*	0.59*	0.17*	1

*p<0.01

**Figure 1:**
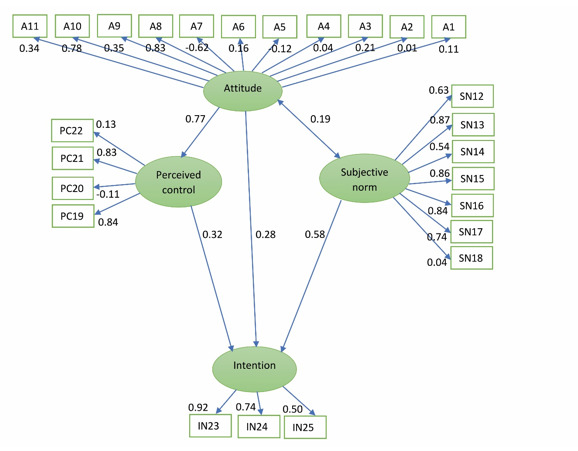
Conceptual diagram of the SEM of the TPB with standard coefficients

 The conceptual diagram of the SEM of the TPB shows the effect of three variables of subjective norms, perceived behavioral control, and attitude on the variable of intention, as well as the effect of questions related to each factor (Figure 1).

According to the results of this model, the two variables of subjective norms and perceived behavioral control have a direct and significant effect on the variable of intention (with *p*<0.001 and *p*=0.015, respectively). The regression coefficients of the paths in Figure 1 indicate that the path of attitude to intention (*p*=0.031, B=-0.22) and the perceived behavioral control (B=1.18, *p*<0.001) are significant. In other words, all direct paths to the intent are statistically significant.

**Figure 2:**
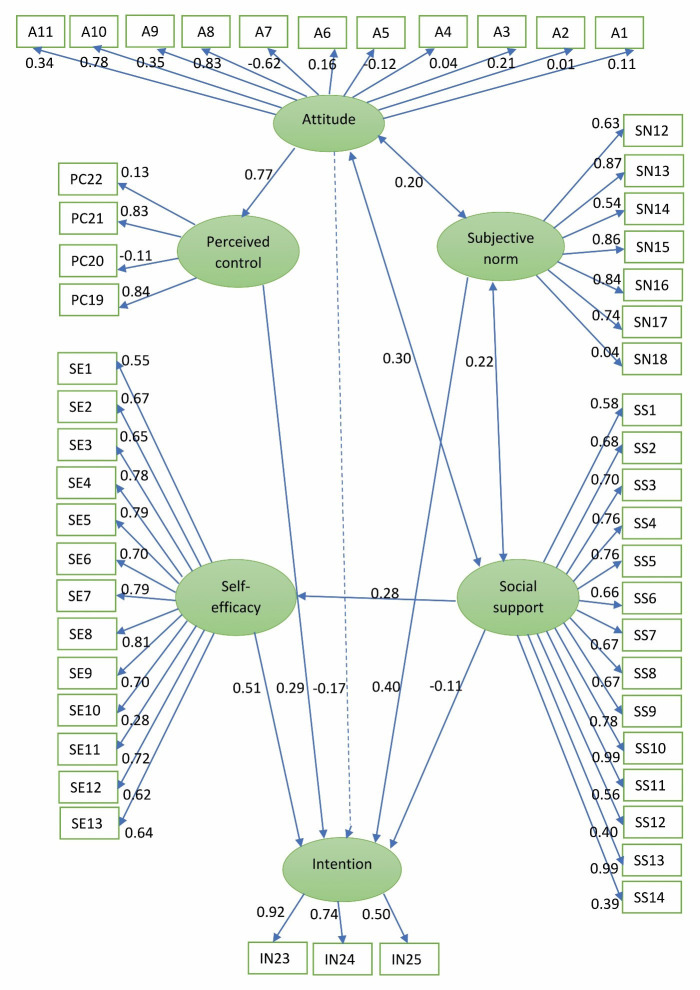
Conceptual diagram of the SEM of the extended TPB along with standard coefficients

To evaluate the goodness of fit of the model, the chi-square to the degree of freedom (DOF) (CMIN / DF) and the RMSEA indices are usually used. In this study, the CMIN / DF index for this model was 2.76, and the RMSEA index was 0.084. Also, the R2 index, or the explained variance for the variable of intention, which represents the percentage of explanation of the changes of the dependent variable (intention) by the independent variables, was calculated as 0.37. In the conceptual diagram of the SEM of the extended TPB, the effect of five variables of self-efficacy, social support, subjective norms, perceived behavioral control, and attitude on the variable of intention as well as the effect of the questions related to each factor are examined and shown (Figure 2).

 According to the results of this model, the three variables of self-efficacy, subjective norms, and perceived behavioral control have a direct and significant effect on the variable of intention (with *p*<0.001, *p*<0.001, and *p*=0.019, respectively).

 The regression coefficients of the paths in Figure 1 indicate that the path of attitude to intention (*p*=0.176, B=-1.32) is not significant, while the path of attitude to perceived behavioral control is significant (*p*<0.001; B=1.20). Also, the path of social support to the intention (*p*<0.110, B=-0.11) is not significant and the path of social support to self-efficacy is significant (*p*<0.001; B=-0.383). Also, the CMIN / DF index for this model was equal to 1.89, and the RMSEA index was equal to 0.060. Also, in this model, the index R2 or the explained variance for the intention variable was calculated as 0.46. 

According to the model introduced in the TPB questionnaire,and extended TPB, the coefficients and the effect of each question on different dimensions are examined, and the final results of these coefficients along with the standard error and the significance level of their effect are presented in Table 2. 

**Table 2:** Coefficients, standard error (SE), and the significance level of constructs in the SEM of the TPB and the SEM of the extended TPB.

Scale	Subscale	Matter	Coefficient	SE	***p*-value**
TPB	Intention	Perceived control	0.165	0.068	0.015
		Subjective Norm	0.524	0.102	<0.001
		Attitude	-0.223	0.103	0.031
	Perceived Control	Attitude	1.182	0.163	<0.001
ETPB	Intention	Perceived Control	0.147	0.063	0.019
		Subjective Norm	0.354	0.081	<0.001
		Self-Efficacy	0.385	0.079	<0.001
		Social Support	-0.110	0.069	0.110
		Attitude	-0.132	0.097	0.176
	Perceived Control	Attitude	1.201	0.166	<0.001

Comparison of two models in estimating intention variable: In this study, two models of structural equations were compared. In the first model (theoretical constructs of planned behavior), the effect of three factors of subjective norms, perceived behavioral control, and attitude on the variable of intention is investigated. In the second model (extended TPB), two factors of self-efficacy and social support have been added to the previous model. According to the goodness of fit indicators reported in Table 3, the extended TPB has a better situation than the TPB in all indicators. Also, the index R^2^ or variance explained for the variable of intention, which indicates that, what percentage of changes in the dependent variable (intention) explained by independent variables, was higher for the second model compared to the first model. This means that the first model explain 37% of the changes in the dependent variable but the second model explains 46% of the changes in the dependent variable.

**Table 3:** Goodness of fit indicators of the two models

CMIN/DF	TLI	PNFI	PCFI	IFI	CFI	RMSEA	R^2^	Index
1-3	>0.90	>0.50	>0.80	>0.90	>0.90	<0.08		Acceptable range
2.756	0.829	0.669	0.721	0.858	0.856	0.084	0.37	Model 1
1.897	0.835	0.671	0.781	0.850	0.848	0.060	0.46	Model 2

## Discussion

Various studies have examined the predictive power of TPB constructs for behavioral intention through some statistical methods, of which some are in line with our study, and some are contrary to our study. In the present study, the extended TPB has been able to explain a higher percentage of changes in the intention variable compared to that in the original model.

In our study, there was no relationship between maternal occupation and the mean scores of EBF, which is consistent with the Haghighi’s,^20^ and Moafi’s studies.^21^ However, although some studies, such as Scott’s study,^22^ consider the occupational conditions of the mother as one of the factors affecting EBF, and the Saffari’s study,^23^ the conditions of EBF may differ in the occupational environment based on the conditions and facilities available for breastfeeding. In the present study, the Pearson correlation coefficient showed a direct and significant correlation between the constructs of the TPB and the constructs of the extended TPB aimed at EBF. Significant correlations in self-efficacy constructs and EBF have been also shown in other similar studies. The Brockway’s study,^13^ showed improved breastfeeding with increased self-efficacy. Brockway^24^ also showed in another study that for each unit of increasing the mean self-efficacy score, the intention to EBF increased by 10%. Although some studies, such as Newhock *et al.*^25^ And Senghore et al 26. Have considered attitude as the most important predictor of intention, the reason for this difference can be related to differences in the social structural elements that have shaped the mothers' lives.

In terms of the predictive power of constructs, if the value of the CMIN / DF index is less than three and the RMSEA index is less than 0.08, it indicates that the model fits well with the data. In our study, the indices of the goodness of fit in both models were acceptable in predicting the intention of EBF, while all indices had a better status in the extended TPB: in the first model, the effect of the attitude variable on both perceived behavioral control variables and intention is significant. Thus, it can be said that the perceived behavioral control variable plays a significant mediating role for the variables of attitude and intention. Considering that the two predictor variables (perceived behavioral control and attitude) can also directly predict the criterion variable (intention) significantly, it can be said that the mediation variable of perceived behavioral control can partially mediate the other two variables. In the second model, the attitude predictor variable cannot predict the intention variable directly, so it can be said that the perceived behavioral control mediator variable is fully capable of meaningful mediation between the two attitude and intention variables. Therefore, the perceived behavioral control construct acts both as a mediating factor (in the first model a partial role and in the extended model a complete role) for the attitude variable and as a direct variable to predict the intent of the behavior.

The R2 index or explained variance for the intention variable in the first model was calculated as 0.37. If this index is less than 0.19, the model is weak. If it is between 0.19 and 0.67, it is medium and if it is more than 0.67, the model will be robust. In this study, the value of this index indicates the average power of the first model. 

In a cross-sectional study conducted by Bazholvand *et al.*,^27^ as in the present study, the goodness of fit indicators of the TPB in predicting the intention of EBF were acceptable, and the perceived behavioral control construct could explain the maximum variance predicting the intention (65%) of EBF. Also in a cross-sectional study aimed at investigating the predictive effect of constructs of the TPB in EBF in primiparous mothers conducted by Jameei *et al.*^28^ Spearman correlation coefficient showed a direct and significant correlation between TPB constructs, and the intention of EBF. Also, the highest correlation was associated with the perceived behavioral control construct. Also, in the extended TPB, the social support predictor variable cannot predict the intention variable directly, but the self-efficacy mediator variable is fully capable of significant mediation between the two variables of social support and intention. 

The results of this study and similar studies^13^,^26^ show that mothers with low levels of self-efficacy are faced with a higher risk of early cessation of breastfeeding. In a study by Dewi Ratnasari et al. the results showed that adequate family support was significantly associated with a higher likelihood of EBF.^29^ (OR: 2.89; 95% CI: 1.29-6.44) In a prospective cohort study conducted by Tengku *et al.*^30^ two hierarchical regression analyzes were performed. The TPB explained 51% of the variance in intention, but adding of the social support construct increased the explained variance by 6%.

The present study is one of the few studies that simultaneously examine the effectiveness of two factors effective on EBF, namely social support and self-efficacy, in addition to the theoretical constructs of planned behavior in the form of SEM. Another strength of the present study is considering the role of the mediating variable, as well as examining the impact of questions related to each factor that has not been considered in the above studies. Therefore, since using the extended model explains a higher percentage of the EBF intention, teaching the skills leading to improved self-efficacy and a sense of empowerment for the intention of EBF and improved family social support, seems necessary in the late pregnancy period.

Our study has some limitations, including that only the intention of EBF has been evaluated. It is suggested that in future studies, EBF behavior will be measured as well to analyze and evaluate the factors affecting the behavior. Given the importance of exclusive breastfeeding in promoting maternal and infant health, and since encouraging and educating mothers is the duty of nurses and midwives working in maternal and infant wards, The use of the structures of this theory can increase the quality and efficiency of nurses' educational interventions, in order to improve the index of exclusive breastfeeding and promote the health of mother and child.

## Conclusion

The results of this study showed that the extension of the TPB and the addition of new constructs to this theory can be appropriate model for predicting the intention of EBF. The present study shows the need for more attention to the implementation of educational programs with optimal impacts on the level of self-efficacy and social support of pregnant mothers, and ultimately the impact on the intention of EBF behavior. It is recommended to conduct several interventional studies to confirm the efficiency of this extended model on promoting the intention and behavior of EBF. 
